# pH-Induced *In Situ* Gel for Periodontal Anesthesia

**DOI:** 10.4103/0250-474X.49120

**Published:** 2008

**Authors:** H. Gupta, R. M. Singh, G. N. Singh, D. Kaushik, A. Sharma

**Affiliations:** Central Indian Pharmacopoeia Laboratory, Raj Nagar, Ghaziabad-201 002, India; 1 University Institute of Pharmaceutical science, Kurukshetra University, Kurukshetra-132 119, India

**Keywords:** *In situ* gel, periodontal anaesthesia, prilocaine hydrochloride

## Abstract

A pH mediated *in situ* gelling system was developed using prilocaine hydrochloride for periodontal anesthesia using combination of chitosan and hydroxypropylmethylcellulose. The gel so developed can be used as anaesthetic in lengthy dental surgery. The gel was evaluated for many parameters like gelation pH, viscosity, physicochemical properties, *in vitro* release, sterility and stability. Gel with chitosan (0.25% w/v) and hydroxypropylmethylcellulose (0.25% w/v) was found to have good gelation near pH 7.4 (pH of mucous) with prolonged action.

It is estimated that approximately 10-30% of the population suffers from periodontal diseases with pathological periodontal pockets. Repeated sub gingival mechanical debriment/cleansing was done to arrest further periodontal tissue destruction. The number of periodontal pockets in a patient may vary as can the pocket depth. Approximately 40% of all periodontal scaling procedures performed involve some kind of anesthesia^1^.

A number of topical anesthetics are used in dentistry, but they have some shortcomings like their low degree of efficacy, tendency to spread in other areas causing numbness of lips and tongue, bitter taste, difficulty in administration, and short duration of action. But as the scaling procedure is unpleasant and painful, anesthetic techniques used are nerve block/infiltration anesthesia in combination with topical anesthesia. So taking into consideration the above shortcomings, this research work was planned to develop a local anesthetic *in situ* gelling system suitable for administering in periodontal pocket, which would enable the patient to have painless treatment without distress of injection, stay at application site due to viscosity increase and give a fast onset of anesthesia lasting throughout the dental procedure and after that can be easily rinsed out with water causing a fast decline in anesthetic effect.

For this a novel polymeric system, gel-forming solution by a simple phase transition (sol-gel transition), mediated by pH were formulated using chitosan and hydroxypropylmethylcellulose (HPMC).

Gufic biosciences Ltd generously gifted prilocaine HCl IP. Chitosan and HPMC were purchased from Sigma-Aldrich, India. All other chemicals used were of analytical grade.

Different combinations of placebo formulations were developed and evaluated for their characteristics. Chitosan (viscosity 175 cps, insolubles 0.20%, deacetylation 84%, pH 8.34, moisture 7%, ash 0.48%, total plate count <5000/G) was dissolved in phosphate buffer system, pH adjusted to 5.5-6.0 by 1% v/v acetic acid. HPMC was dissolved in phosphate buffered saline, pH 7.4. Viscosity was measured using Brookfield`s viscometer (model DV II, spindle no.02, at 20rpm), while clarity was examined through visual inspection. Different combinations tested are shown in Table 1. The formulations are selected/rejected on the basis of their clarity, turbidity and viscosity with change in pH.

**TABLE 1:** PYSICOCHEMICAL CHARACTERIZATION OF PLACEBO FORMULATIONS

Formulation	Chitosan (%w/v)	HPMC (%w/v)	Physical characteristic		Viscosity (in cps)	
				
Code			At pH 6.0	At pH 7.4	At pH 6.0	At pH 7.4
F1	0.5	-	Clear solution	Turbid solution	33±2	77±1.5
F2	0.4	0.1	Clear solution	Slight turbid solution	35±1	70±2
F3	0.3	0.2	Clear solution	Slight turbid solution	36±1.2	62±1.8
F4	0.25	0.25	Clear solution	Very slightly turbid	36±1.5	55±2
F5	0.15	0.35	Clear solution	Clear solution	37±1	58±1.4
F6	0.1	0.4	Clear solution	Clear solution	38±2.2	47±2
F7	-	0.5	Clear solution	Clear solution	40±1.7	40±2

All values are expressed as mean±SD (n=6)

From the above data formulation F4 consisting of chitosan/HPMC (0.25% each) was found to be optimum and was used for further studies. However formulations F1 (chitosan alone, 0.5%) and F7 (HPMC alone, 0.5%) were also studied to serve as control for comparison. For periodontal anesthetic activity prilocaine HCl was prescribed in a dose of 5%. Hence a dose of 5% was used during formulation. Complete formulae for different formulations were shown in Table 2.

**TABLE 2:** INGREDIENTS OF MEDICATED FORMULATIONS

Ingredients	F1	F4	F7
Prilocaine HCl	5%	5%	5%
Chitosan	0.5%	0.25%	-
HPMC	-	0.25%	0.5%
Methylparaben	0.1%	0.1%	0.1%
Water (qs)	100%	100%	100%

Medicated formulations F1, F4 and F7 were tested for their physicochemical characteristics i.e. clarity, gelation pH, viscosity and refractive index. The clarity of the formulations after and before gelling was determined by visual examination of the formulations under light alternatively against white and black backgrounds. Gelation pH is the pH at which the solution form of the formulation was changed to gel. Formulation was taken in a beaker and 1M NaOH was added dropwise with continuous stirring, pH was checked using pH meter The pH at which sudden change in viscosity was observed and noted as gelation pH (Table 3). Viscosity of formulations were determined by using Brookfield's viscometer (model DV II, spindle no. 02, at 20 rpm), on formulation pH 6.0 and gelation pH near 7.4 (Table 3). Refractive index of the formulations F1, F4 and F7 were determined with Abbe`s refractometer. Data is shown in Table 3.

**TABLE 3:** PHYSICOCHEMICAL PROPERTIES OF VARIOUS FORMULATIONS

Codes	Clarity	Gelation pH	Refractive index	Viscosity (in cps)	
				
				At pH 6.0	At pH 7.4
F1	Clear solution	6.9±0.2	1.332±0.001	33±1.2	77±2.4
F4	Clear solution	7.1±0.1	1.334±0.001	36±1.1	75±3.7
F7	Clear solution	-	1.337 ±0.002	40±2.3	40±3.2

All values are expressed as mean±SD (n=6)

Medicated formulations, F1, F4 and F7 screened out from the rheological studies and physical characterization were subjected to *in vitro* release studies using the dialysis technique. One millilitre formulation was taken in the dialysis tubes (molecular weight cut off 12, 000, Sigma Inc, MO, USA), which was suspended in beaker at 37±0.5° containing 100 ml simulated fluid (sodium chloride 0.670 g, sodium bicarbonate 0.200 g, calcium chloride dihydrate 0.008 g, and purified water q.s. 100 g), pH 7.4 under continuous stirring. One millilitre of samples was withdrawn at different time intervals and equal volumes of fresh media were added to replace the withdrawn samples. Withdrawn samples were diluted appropriately and absorbances of the samples were determined by UV spectrophotometrically at λ_max_ 230 nm. Cumulative percent drug released was calculated. The optimized formulations were packaged in amber colored bottle fitted with a teat and sterilized by autoclaving at 121° at 15 lb/inch^2^ gauge pressure for 20 min. The tests for sterility were also conducted on these formulations as per Indian Pharmacopoeia^3^.

The international conference on harmonization (ICH) Tripartite Guidelines defines the stability testing. Three packs of preparation F4 packed in amber colored bottle sealed with rubber teat were subjected to stability studies. The samples were kept in humidity and temperature control cabinet (Topsun, China) with automatic control to maintain constant temperature and humidity. The cabinet was then set at 40° temperature and 75% relative humidity. At 0, 30, 60 and 90 days 0.1 ml of samples were withdrawn, diluted with distill water to 10 ml and assayed by UV at 230 nm. Samples were also tested for viscosity, gelation pH and appearance after every one month till three months.

Present study employed combination of chitosan and HPMC for the development of periodontal anesthesia. Properties of chitosan like bioadhesiveness, viscous nature and ability to convert into hydrogel at mucus pH (pH 7.4) make it best suitable candidate for the development of such type of delivery systems. It was observed from the results that on increasing the concentration of HPMC imparts clarity to the formulation without affecting its viscosity. When the two polymers were mixed in equal concentration (0.25% each) the formulation become very slight turbid at pH 7.4. Chitosan is insoluble at neutral or alkaline pH and converted in to gel when the pH of the formulation is raised and resulted in sudden increases in the viscosity.

*In vitro* drug release profile of the formulation was determined in simulated fluid (pH 7.4) and the formulation displayed a slow release profile (fig. 1). Chitosan/HPMC based formulation (F4) displayed a 28.43% cumulative drug release after 2 h, 42.44% after 6 h and 96.27% after 24 h. More or less similar pattern was observed with pure chitosan based formulation (F1). Pure HPMC based formulation (F7) exhibited faster release as compared to chitosan based formulations. A cumulative 98.03% release was observed in 10 h from the pure HPMC based formulation (F7).

**Fig. 1:**
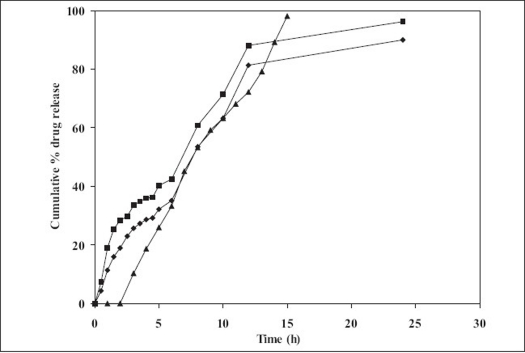
*In vitro* drug release from various developed formulation. Chitosan formulation F1 (–♦–), chitosan and HPMC F4 (–■–) and HPMC formulation F7 (–▲–)

For packing the formulations the amber colored bottle closed with rubber closure and dropper with teat were used and found to be appropriate packaging system for current formulation. The packaging material was tested for resistance for autoclaving, leakage and pourability. Packages passed all the tests and proved to be a good choice for packaging of present formulation.

In sterility testing, no microbial growth/ turbidity was observed up to 14 days of incubation at 30° to 35° in case of fluid thioglycolate medium and at 20° to 25° in the case of soyabean-casein digest medium. Hence the formulation passed the sterility test. According to ICH guidelines for stability studies, graph was plotted between time (in days) and drug remaining in pack. Degradation constant (K) was calculated from the slope of line, which was found to be 3.07×10^−4^ days^−1^. As the amount of drug degraded in 90 days was 3.243%; therefore arbitrary shelf life of 2 y was assigned to the optimized formulation as the amount of degradation was less then 5%.

Studies revealed that the pH mediated *in situ* gelling system can be formulated using optimum concentration of chitosan and HPMC that can gel at mucus pH with prolong action of prilocaine HCl that may be helpful for periodontal anaesthesia, where lengthy procedure is required.
